# Transcriptional Analysis of *Chlorella pyrenoidosa* Exposed to Bisphenol A

**DOI:** 10.3390/ijerph16081374

**Published:** 2019-04-16

**Authors:** Leyi Duan, Qi Chen, Shunshan Duan

**Affiliations:** Department of Ecology, College of Life Science and Technology, Jinan University, Guangzhou 510632, China; 343363000luoluo@sina.com (L.D.); cq92088@outlook.com (Q.C.)

**Keywords:** Bisphenol A (BPA), *Chlorella pyrenoidosa*, inhibitory effect, stimulation effect, transcriptome analyse

## Abstract

Bisphenol A (BPA) is the raw material of 71% of polycarbonate-based resins and 27% of epoxy-based resins which are used for coating metal-based food and beverage cans. Meanwhile, it is taken into account as a typical environmental pollutant. Hormesis may occur in algae exposed to BPA. In this study, the effects of BPA on *Chlorella pyrenoidosa* were assessed based on growth inhibition and transcriptome analysis. We have focused on two exposure scenarios as follows: (1) exposure to a low stimulation concentration (0.1 mg.L^−1^, 19.35% promotion in cell density on the 3rd day); (2) exposure to a high inhibition concentration (10 mg.L^−1^, 64.71% inhibition in cell density on the 3rd day). Transcriptome analysis showed enrichment in nucleotide transport, single-organism transport, cellular respiration. Among them, adenosine triphosphate (ATP) synthase and Nicotinamide adenine dinucleotide (NADH) dehydrogenase were upregulated under 0.1 mg.L^−1^ BPA treatment. These changes enhanced the physiological and energy metabolic pathways of *C. pyrenoidosa*, thereby stimulating cell proliferation. At exposure to the high BPA, severe inhibited changes in the expression levels of several pathways were observed, which were related to tricarboxylic acid (TCA) cycle, glycolysis, fatty acid metabolism, oxidative phosphorylation, and photosynthesis. Therefore, BPA could negatively affect growth inhibition through the multiple energy metabolism processes. These results may result in a deeper insight into BPA-induced biphasic responses in algae, and provide vital information to assess the potential ecological risks of exposure to BPA in an aquatic ecosystem.

## 1. Introduction

Bisphenol A (BPA) is an environmental endocrine-disrupting chemical (EDC), associating with the estrogen effect, that is widely utilized in industrial applications [1]. In addition, BPA disrupts the homeostasis of internal environment and affects the growth and development of organisms by interfering with the normal synthesis of hormones in organisms [2]. BPA is considered to be associated with diabetes, obesity, cardiovascular disease, reproductive diseases, breast cancer. [3,4]. Global production of BPA has exceeded 6 million pounds (lbs) since 2003 [5]. Nowadays, pollution by BPA almost exists to varying degrees throughout the world. Moreover, its production rate account for about 43.5% of the gross world production, and the trend tends to increase [6]. In recent years, due to the intensification of industrial activities, a great number of BPA have been discharged into the water body and transmitted through the food chain in an aquatic ecosystem, seriously threatening the safety of drinking water and the balance of the aquatic ecosystem [7].

Algae are the most important primary producers in the aquatic ecosystem, playing a pivotal role in maintaining the ecological balance [8]. In addition, algae are also able to accumulate highly toxic substances (e.g., selenium, zinc, and arsenic) in their cells and/or bodies, thereby eliminating such substances from an aquatic environment [9]. Recently, the impact of BPA on algae has significantly attracted scholars’ attention. Exposure to high concentration of BPA may inhibit the growth of algal cells [10]. However, BPA also acts as a hormetic substance at low concentration. Hormesis refers to a biphasic dose-response relationship characterized by stimulation at low doses and inhibition at high doses [11]. To date, it has been reported that low concentration of toxic or organic substances could stimulate the growth of microalgae, and this phenomenon might contribute to the outbreak of algal blooms to some extent [12,13]. Hence, it is necessary to elucidate the molecular mechanism of hormesis. The majority of previous studies have mainly focused on how BPA might affect the physiological indicators (e.g., growth rate, chlorophyll synthesis) in algae, while no in-depth study on toxicological mechanism has been carried out yet [14,15]. Moreover, a limited number of studies have concentrated on the hormetic mechanism of BPA or other organic pollutants, and the inhibitory effects have been mainly studied. To date, with the development of RNA sequencing (RNA-Seq), the recognition of molecular level of eukaryotic organisms has been continuously improved [6]. Fan et al. [16] conducted a transcriptome-based analysis on gene expression of *C. pyrenoidosa* under different CO_2_ concentrations, and found that the CO_2_-concentrating mechanism was activated at low CO_2_ environment to compensate for the low activity of RuBisCO in the Calvin cycle. Qian et al. [17] investigated the inhibitory effects of linoleic acid (LA) on *C. pyrenoidosa* by transcriptome analyses, and reported that genes related to photosynthesis, carbon metabolism, and amino acid metabolism were inhibited, which could be key targets for LA in *C. pyrenoidosa*. Cheng et al. [18] performed RNA-Seq to justify why astaxanthin yield of the *Haematococcus pluvialis* mutant was 1.7 times higher than that of a wild strain, owning to the enhancement of pyruvate and fatty acid metabolism for supporting biosynthesis of astaxanthin. Therefore, the hormetic mechanism of BPA needs to be effectively studied.

*Chlorella pyrenoidosa* is one of the most representative green algae, associating with strong adaptability, rapid reproduction, and sensitivity to pollutants. To date, the hormetic mechanism of BPA on algae (e.g., *C. pyrenoidosa*) has been rarely studied. In this study, we aimed to apply RNA-Seq to disclose the hormetic mechanism of green algae.

## 2. Materials and Methods

### 2.1. Algal Strains and Growth Conditions

Here, *C. pyrenoidosa* was provided by theResearch Center of Hydrobiology, Jinan University, Guangzhou, China, which was isolated from a freshwater sample. The algae cells were grown in a flask, containing 100 mL of BG11 medium. Algal fluid was grown in an artificial climate box (CC275TL2H; Hangzhou Xutemp Temptech Co., Ltd., Hangzhou, China). The light intensity was 1200 lux, with the temperature of 25 ± 2 °C under 12:12 h light-dark cycle. The flask was shaken three times/day, and the conical flask was changed randomly to ensure that the microalgae are exposed to the uniform light in the conical flask.

### 2.2. Experimental Process

Here, BPA was dissolved into methanol, and it was essential to ensure that the concentration of methanol did not exceed 0.5%. According to a previous experiment [19], under this threshold, the toxicity of methanol would be negligible. We incubated 100 mL of algal culture in 150-mL flask for subsequent experiments for 5 days. The initial cultivated density of algae in exponential growth phase was 2.8 × 10^5^ cells/L. In order to investigate the effects of BPA on *C. pyrenoidosa*, different concentrations of BPA (0, 0.1, 1, and 10 mg. L^−1^) were added into algal culture. The experiments were conducted in triplicate. The flasks were shaken three times/day, and changed randomly to ensure that the microalgae exposed to uniform light in the flask.

To verify changes at transcription level, samples treated for 72 h by BPA at concentrations of 0, 0.1, and 10 mg.L^−1^ were selected. Furthermore, 100 mL of *C. pyrenoidosa* was centrifuged at 8000 rpm for 5 min, and then supernatant and extracted cell pellets were removed.

### 2.3. Growth and Chlorophyll Fluorescence Analysis

The cell density was daily measured by a flow cytometer (BD Accuri C6, Becton Dickinson, Franklin Lakes, NJ, USA). A chlorophyll fluorometer (TD700; Turner Design, Inc., Chicago, IL, USA) determined the content of chlorophyll a (Chla). Maximum quantum efficiency of PSII photochemistry (Fv/Fm) is a common parameter. Under normal conditions, Fv/Fm is extremely stable; when algae or plants are stressed, Fv/Fm significantly decreases. Therefore, Fv/Fm is an important index to study the influences of various stresses on photosynthesis [20]. The Fv/Fm was detected by a portable plant efficiency analyzer (PEA; Hansatech Instruments Ltd., Norfolk, UK). The cultivation of the algae was carried out at dark for 20 min before measurement at room temperature (26 ± 1 °C).

### 2.4. RNA Extraction and cDNA Library Construction

Total RNA was extracted by TRIzol reagent (Invitrogen, Carlsbad, CA, USA) on the basis of the manufacturer’s instructions. After that, oligo (dT) magnetic beads were highly enriched with mRNA. Second-strand cDNA was then synthesized for 2 h at 16 °C using the total 20 µL first-strand product plus 80 µL of second strand mix containing 63 µL water, 10 µL second-strand buffer, 4 µL dNTPs, 2 µL DNA Polymerase and 1 µL of RNase H. Then, the cDNA fragments were purified with a QIAquick Polymerase Chain Reaction (PCR) Purification Kit (Qiagen, Hilden, Germany). The ligation products were selected by agarose gel electrophoresis, PCR amplified, as well as sequencing using Illumina HiSeq^TM^ 2000 (Illumina, Inc., San Diego, CA, USA).

### 2.5. Analysis of Differentially Expressed Genes

To identify differentially expressed genes across samples or groups, the edgeR package (http://www.r-project.org/) was used. Differentially expressed genes (DEGs) were further annotated to Gene Ontology (GO) database and Kyoto Encyclopedia of Genes and Genomes (KEGG) database.

### 2.6. Quantitative Reverse Transcription Polymerase Chain Reaction (RT-qPCR)

Quantitative reverse transcription polymerase chain reaction (RT-qPCR) was carried out to confirm the expression of *C. pyrenoidosa* gene profiles. RNA samples extracted from three biological replicates exposed to 0.1, 1.0, and 10 mg.L^−1^ BPA were analyzed by RT-qPCR. In addition, RT-qPCR was conducted using a SYBR Green Master kit (Takara, Tokyo, Japan) in accordance with manufacturer’s protocol, and the results were normalized by 18S rRNA. The primers were designed according to the sequence of the transcriptome Denevo. Primers used in RT-qPCR are listed in Table 1.

### 2.7. Statistical Analysis

All experiments were repeated three times independently. Data were analyzed using one-way analysis of variance (ANOVA) with the help of SPSS 13.0 software (IBM, Armonk, NY, USA), and were presented as the mean ± standard deviation (SD). The significance level of differences was set at *p* < 0.05.

## 3. Results

### 3.1. Effects of Bisphenol A (BPA) on Growth and Photosynthetic Efficiency of Algae

**Figure 1:**
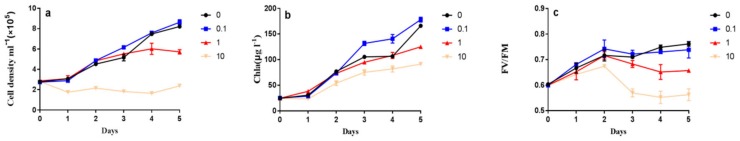
*C. pyrenoidosa* in response to bisphenol A (BPA). (**a**) cell density, (**b**) the content of Chla, (**c**) photosynthesis activity.

As shown in Figure 1a, on the first two days, no significant effects on growth of algae were observed after exposure to 0.1 and 1 mg.L^−1^ (*p* > 0.05). When the BPA concentration reached 10 mg.L^−1^, cell density was lower than that in other therapeutic groups. On the 3rd day, the cell density in the therapeutic groups of 0.1 and 10 mg.L^−1^ treatment groups was significantly increased (*p* < 0.001) by 19.35% and decreased (*p* < 0.001) by 64.71% compared with the control group, respectively. The results for Chla content (Figure 1b) showed a similar trend compared with the cell density. The Chla content of algae treated with 0.1 mg.L^−1^ BPA was markedly increased (*p* < 0.001) from the 3rd day. Obvious promotion occurred on the 3rd day of exposure to BPA, and the Chla content on the 3rd day was increased (*p* < 0.001) by 24.94% of the control group at concentration of 0.1 mg.L^−1^. In addition, with respect to the control group, at a high-dose of exposure to BPA (10 mg.L^−1^), the content of Chla was inhibited (*p* < 0.001). The change of Fv/Fm value was given in Figure 1c, and *C. pyrenoidosa* cells treated for 2 days showed a slightly promotion of low concentration and inhibition of high concentration.

### 3.2. Global Transcriptional Changes of C. pyrenoidosa

**Figure 2:**
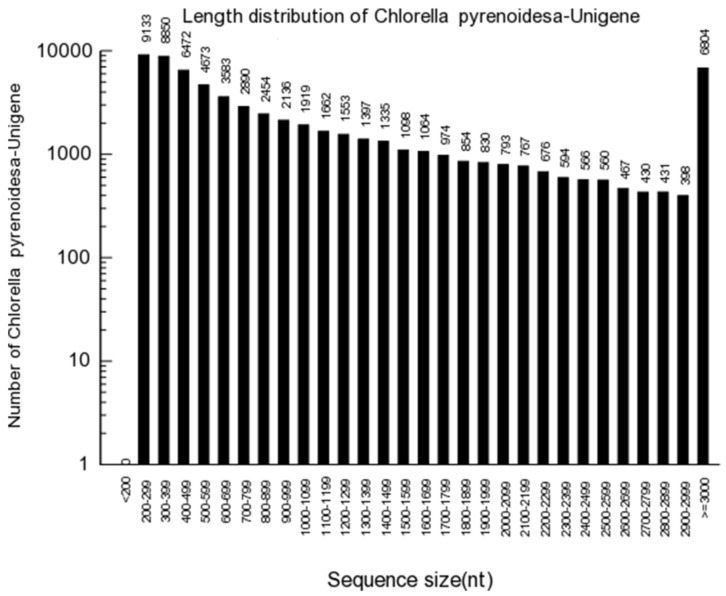
The length of distribution of unigens. The x-axis shows the scope of the sequence size.

**Figure 3:**
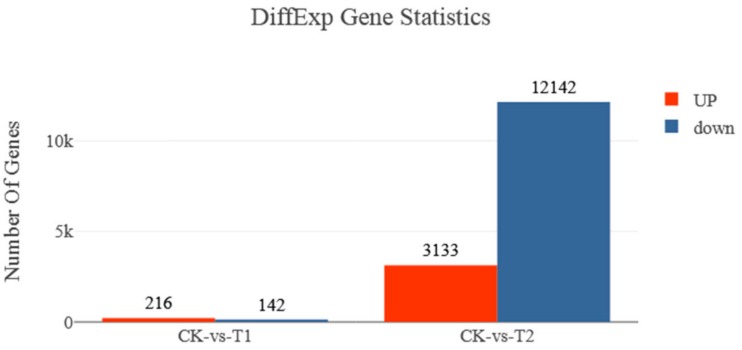
The number of differentially expressed genes (DEGs) at two groups compared with that at CK group. CK represents Control group, T1 represents 0.1 mg.L^−1^ BPA treatment group, and T2 shows 10 mg.L^−1^ BPA treatment group.

A total of 65,363 unigenes with 2298-bp of unigene N50 were obtained. The distribution of unigenes is illustrated in Figure 2. A false discovery rate (FDR) ≤0.05 and |log2| ≥1 were used as threshold to judge the significance of DEGs, as shown in Figure 3. After exposure to 0.1 mg.L^−1^ BPA, 216 genes were upregulated, whereas 142 genes were downregulated. At the same time, 10 mg.L^−1^ treatment group showed that 3133 genes were upregulated, while 12,142 genes were downregulated.

### 3.3. Differentially Expressed Genes (DEGs) Exposed to Low Concentration of BPA

**Figure 4:**
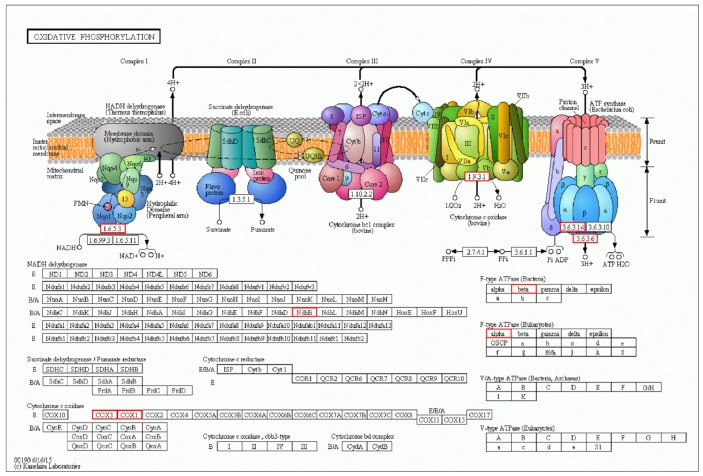
Differentially expressed genes related to oxidative decarboxylation in *C. pyrenoidosa* under CK (control group) and T1 BPA (red color: upregulated genes).

Results of GO enrichment analysis are presented in Table 2; no downregulated GO term was significantly enriched in low concentration of BPA (T1 represents 0.1 mg.L^−1^ BPA treatment group), while the GO term enrichment of gene was upregulated in T1, the terms “single-organism transport (GO:0044765)” and “single-organism localization (GO:1902578)” were sigificantly overrepsented, as well as the enrichment of terms “nucleoside transport (GO:0006810)” and “cellular respiration (GO:0045333)”. The KEGG database was also used to analyze pathways, in which DEGs were involved as well (Table 3). Besides, T1 affected ribosomes, oxidative phosphorylation, and sulfur metabolism. In oxidative phosphorylation, the DEGs were all upregulated, including *ATPF1B*, *COX1*, *COX3*, and *ndhB* (Figure 4).

### 3.4. DEGs Exposed to High Concentration of BPA

At higher BPA concentration, the cell projection (GO:0042995) and tetrapyrrole metabolic process (GO:0033013) were significantly upregulated, while branched-chain amino acid metabolic process (GO:0009081) and kinase activity (GO:0016301) were downregulated. Meanwhile, amino sugar and nucleotide sugar metabolism, photosynthesis-antenna proteins, and fatty acids were enriched in T2 (T2 represents 10 mg.L^−1^ BPA treatment group). In T1, upregulated genes were more frequent. By contrast, downregulated genes were predominant in T2. In other metabolic pathways, the expression of genes was significantly downregulated (Table 3).

### 3.5. RT-qPCR for Analysis of Expression of Related Genes

**Figure 5:**
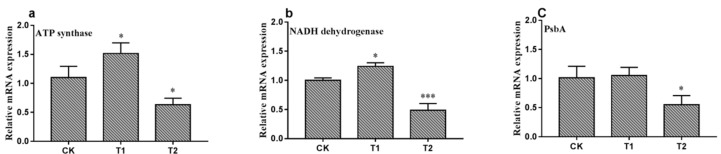
The mRNA expression of ATP synthase (**a**), NADH dehydrogenase (**b**), and PsbA (**c**) in *C. pyrenoidosa* exposed to 0.1 mg.L^−1^ BPA (T1) and 10 mg.L^−1^ BPA (T2). *C. pyrenoidosa* without exposure to BPA were used as control group. The data were expressed as mean ± standard deviation (SD) compared with control group * *p* < 0.05 vs. control group, *** *p* < 0.001 vs. control group

The relative mRNA expression level of several genes after exposure to 0.1 and 10 mg.L^−1^ BPA were detected by RT-qPCR (Figure 5). The expression levels of ATP synthase and NADH dehydrogenase were significantly upregulated at exposure to 0.1 mg.L^−1^ BPA. In contrast, at 10 mg.L^−1^ BPA group, the expression levels of three genes were significantly decreased. This result indicates that synthesis of ATP is stimulated at low concentration level, and BPA at high concentration level is harmful to mitochondria and photosynthesis.

## 4. Discussion

### 4.1. Effects of BPA on Growth Inhibition

In the present study, in the highest concentration, the cell density, Chla content, and Fv/Fm were all decreased, and the growth was inhibited, which was consistent with Zhang et al.’s results [21]. Photosynthesis is sensitive to toxicity [22], thus we speculate that growth inhibition might be partly related to disturbance of Chla, in addition to synthesis of photosythesis. At 0.1 mg.L^−1^ BPA treatment group, the growth of *C. pyrenoidosa* and the content of Chla were stimulated. The stimulation response to BPA concentration is similar to observation of another algae, *Stephanodiscus hantzschii* [23], in which at 3 mg.L^−1^ BPA treatment, growth of algae was slightly stimulated. In addition, a hormetic effect on algae was also found in other EDCs or organic pollutants [24,25]. In brief, BPA is toxic or hormetic substance depending on its concentration.

### 4.2. Molecular Response to Low Concentration of BPA

In this study, the low concentration of BPA increased the growth of *C. pyrenoidosa*, and at the molecular level, the stimulating effect of BPA by promoting energy metabolism was observed. Electron transport chain and oxidative phosphorylation are the most critical reactions in life-sustaining activities, in which the absence of one can affect cellular respiration and even lead to cell death [26]. Mitochondrial electron transport chain, as an important component of mitochondrial structure, plays a exemplary role in regulating cell proliferation and apoptosis [27,28]. The crucial process of oxidative decarboxylation mainly occurs in electron transport chain. The gene *ATPF1B* encoded ATP synthase subunit beta is important cellular oxidative phosphorylation, regulating the rate of ATP synthesis in eukaryotic cells [29]. ATPase activity has been previously considered to be closely associated with cell proliferation and growth, and the technology of ATP bioluminescence for cell proliferation and determination of cytotoxicity has been developed for a long-time [30]. The upregulation of *ATPF1B* means the process of the synthesis of ATP from adenosine diphosphate (ADP), in which the content of inorganic phosphate was increased. The increased intracellular ATP promotes the activation of cell-cycle regulators and promotes cell proliferation as well [31]. This finding was similar to Pillai et al.’s result, in which it was revealed that low concentration of Ag^+^ could stimulate ATP synthesis in *Chlamydomonas reinhardtii* [32]. NADH dehydrogenase is the largest and most complex enzyme in mitochondrial proton transferase. It catalyzes oxidation of NADH to NAD^+^ and transfers electron to ubiquinone [33]. The upregulation of NADH dehydrogenase (EC:1.6.5.3) accelerated the rate of electron transport in respiratory chain. The other DEGs in oxidative phosphorylation were upregulated as well. These results showed the increase of ATP synthesis. The ATP synthesis was also affected the level of nucleotide, which were found to be essential for cell proliferation [34]. In GO term related to nucleotide, transport was significantly upregulated. Consistent with these results, the GO term related to ribosome was also upregulated, and it was previously noted that ribosome plays a significant role in proliferation of cells [35]. Briefly, the data showed that the rate of cell proliferation was accelerated and energy and materials were provided for the generation of cell stimulatory effects as well.

### 4.3. Molecular Response to High Concentration of BPA

Glycolysis, tricarboxylic acid (TCA) cycle, and mitochondrial electron transport chain are essential for energy provision in physiological functions [36]. The *gltA* encoded citrate synthase, that is a key point to control the speed of metabolism and the rate of turnover of the TCA cycle in some conditions, was downregulated (Table 4) [37]. Meanwhile, *IDH1*, *IDH2*, and *IDH3* encoded isocitrate dehydrogenase, contributing to oxidative decarboxylation of isocitric acid to produce α-ketoglutarate (α-KG) in TCA, were also downregulated [38]. A downregulation of the genes indicated the production of α-KG and acetyl-CoA was suppressed in *C. pyrenoidosa*, and the process of the TCA cycle was inhibited as well. The TCA cycle is a metabolic pathway of three major nutrients (carbohydrates, lipids, and amino acids), and intermediate products include raw materials for the synthesis of carbohydrates, lipids, and amino acids [39]. The restriction of the TCA cycle associated with sugars, lipids, and amino acids are not fully oxidized to obtain energy, and their synthesis is also affected. Several genes associating with glycolysis were downregulated, demonstrating similar results for TCA cycle.

In fatty acids metabolism, acetyl-CoA is catalyzed to form malonyl-CoA [40]. Acetyl-CoA carboxylase is inhibited, thereby reducing the carboxylation of acetyl-CoA to malony-CoA. The fatty acid metabolism is inhibited in some extent as well. The process of energy acquisition by *C. pyrenoidosa* through oxidative decomposition of sugars and nutrients is inhibited in presence of exposure to high concentration of BPA [41].

In our study, mitochondrial function was also damaged via exposure to high concentrations of BPA. Similarly, Jiang et al. found that the mitochondrial membrane potential and the expression of some oxidative phosphorylation-related genes were downregulated even during exposure to 50 μg/kg/day BPA in rats [42]. The injury to mitochondria indicated that ATP synthesis decreased, and the algae cells could not obtain enough energy to maintain cell survival.

## 5. Conclusions

In the present study, a novel framework for physiological and molecular responses of *C. pyrenoidosa* to BPA was presented. Treatment under 0.1 mg.L^−1^ BPA led to slight growth stimulation. The energy metabolic and nucleotide pathways enhancing provided more energy and material for the promotion of *C. pyrenoidosa*. At exposure to 10 mg.L^−1^ BPA, the repression of genes associated with the TCA cycle, glycolysis, fatty acid metabolism, and mitochondrial electron transport could be observed, suggesting that the BPA inhibits growth of algal through multiple pathways. In a word, BPA is a toxic or hormetic agent to *C. pyrenoidosa* depending on concentration, thereby inhibiting or stimulating their growth through regulating energy metabolism.

## Figures and Tables

**Table 1:** Primer used in quantitative reverse transcription polymerase chain reaction (RT-qPCR) analysis.

Gene Name	Primer Sequence (5′–3′)
18S (Control)	F:TGGTGCCCTTCCGTCAAT
	R:CGGCACCTTACGAGAAATCA
Adenosine triphosphate (ATP) Synthase	F:GAAGTCGGCAATGGTGTCC
	R:CTGGGAGATGAGCACTACGG
Nicotinamide adenine dinucleotide (NADH) dehydrogenase	F:ATCAAGGAAAAGCAGGGGCA
	R:TGTCCCAAAGCATGAAGGCA
*PsbA* (photosystem II D1 protein)	F:TGAACGAGAGTTGTTGAAAGAAGC
	R:TGCTTGGTGTTGCTGGTGTA

**Table 2:** Results of Gene Ontology (GO) enrichment analysis.

GO ID	Description	*p*-Value
T1-UP		
GO:0044765	single-organism transport	0.0015563
GO:1902578	single-organism localization	0.0017646
GO:0006810	transport	0.0020785
GO:0005840	ribosome	0.0022349
GO:0016843	amine-lyase activity	0.0049691
GO:0015858	nucleoside transport	0.0052014
GO:0005198	structural molecule activity	0.0071512
GO:0045333	cellular respiration	0.007919
GO:0051234	establishment of localization	0.0081377
**T2-UP**		
GO:0042995	cell projection	3.81 × 10^−^^6^
GO:0033013	tetrapyrrole metabolic process	1.18 × 10^−5^
GO:0051186	cofactor metabolic process	2.35 × 10^−5^
GO:0098796	membrane protein complex	2.56 × 10^−5^
GO:0006778	porphyrin-containing compound metabolic process	3.79 × 10^−5^
GO:0044422	organelle part	9.72 × 10^−5^
**T2-DOWN**		
GO:0009081	branched-chain amino acid metabolic process	0.0059409
GO:0016301	kinase activity	9.19 × 10^−^^6^
GO:0036094	small molecule binding	0.0001015
GO:0016773	phosphotransferase activity, alcohol group as acceptor	0.0001521
GO:0016772	transferase activity, transferring phosphorus-containing groups	0.0003224
GO:0001883	purine nucleoside binding	0.000369
GO:0032549	ribonucleoside binding	0.000369
GO:0032550	purine ribonucleoside binding	0.000369
GO:0019899	enzyme binding	0.0004276
GO:0004672	protein kinase activity	0.0004357
GO:0017016	Ras GTPase binding	0.0004891
GO:0031267	small GTPase binding	0.0004891
GO:0051020	GTPase binding	0.0004891
GO:0001882	nucleoside binding	0.0005225
GO:0097367	carbohydrate derivative binding	0.0006553
GO:0031981	nuclear lumen	0.0092762
GO:0043233	organelle lumen	0.0092762
GO:0070013	intracellular organelle lumen	0.0092762

**Table 3:** The upregulated and downregulated genes within the Kyoto Encyclopedia of Genes and Genomes (KEGG).

Pathway	Upregulated	Downregulated	*p*-Value
T1vsCK			
Ribosome	11	2	0.000351118
Oxidative phosphorylation	8	/	0.001435787
Sulfur metabolism	4	/	0.002234359
Propanoate metabolism	3	1	0.008392114
Carbon metabolism	8	1	0.01697323
Sulfur relay system	1	1	0.03425709
Cysteine and methionine metabolism	4	/	0.03562303
Microbial metabolism in diverse environments	9	1	0.03764153
Biosynthesis of antibiotics	10	1	0.0470081
**T2vsCK**			
Amino sugar and nucleotide sugar metabolism	3	59	9.38 × 10^−5^
Photosynthesis-antenna proteins	21	22	0.000106969
Fatty acid degradation	13	30	0.000759285
Galactose metabolism	10	26	0.003986388
Microbial metabolism in diverse environments	58	213	0.004257043
Biosynthesis of unsaturated fatty acids	9	26	0.007765076
Valine, leucine and isoleucine degradation	12	44	0.008513731
Ether lipid metabolism	5	15	0.008521474
Fructose and mannose metabolism	13	29	0.00854504
Biosynthesis of antibiotics	55	259	0.009106183
Fatty acid metabolism	21	47	0.01303567
Valine, leucine and isoleucine biosynthesis	1	31	0.01553805
DNA replication	5	52	0.0200779
Metabolic pathways	204	919	0.02039731
alpha-Linolenic acid metabolism	6	19	0.02238955
Sphingolipid metabolism	7	25	0.0240593
Biosynthesis of secondary metabolites	117	416	0.03031573
Peroxisome	18	62	0.0312795
Carbon metabolism	46	151	0.04453267
Nitrogen metabolism	6	19	0.04458744
Pyruvate metabolism	18	57	0.04607645

**Table 4:** Annotation and expression changes of unigenes related to the Tricarboxylic acid (TCA) cycle, glycolysis, oxidative phosphorylation, and fatty acid metabolism.

K-ID	Gene ID	Annotation	log2 Ratio	*p*-Value
Citrate Cycle (TCA Cycle)				
K00030	0047088	IDH3; isocitrate dehydrogenase (NAD+)	−1.33141	6.5 × 10^−7^
K00031	0059207	IDH1, IDH2, icd; isocitrate dehydrogenase	−1.31562	4.1 × 10^−14^
K00161	0045616	PDHA, pdhA; pyruvate dehydrogenase E1 component alpha subunit	−8.30485	0.00024
K00162	0053032	PDHB, pdhB; pyruvate dehydrogenaseE1 component beta subunit	−1.17676	1.4 × 10^−8^
K01647	0010385	CS, gltA; citrate synthase	−1.81821	5.8 × 10^−11^
**Glycolysis**				
K00134	0018211	GAPDH, gapA; glyceraldehyde 3-phosphate dehydrogenase	−2.95214	0.01767
K00850	0047002	pfkA, PFK; 6-phosphofructokinase 1	−1.51129	7.6 × 10^−10^
**Oxidative phosphorylation**				
K00234	0003771	SDHA, SDH1; succinate dehydrogenase (ubiquinone) flavoprotein subunit	−1.06437	1.1 × 10^−11^
K00235	0044774	SDHB, SDH2; succinate dehydrogenase (ubiquinone) iron-sulfur subunit	−1.01169	2.3 × 10^−8^
K02256	0064509	COX1; cytochrome c oxidase subunit 1	−5.49004	1.2 × 10^−10^
K02261	0028443	COX2; cytochrome c oxidase subunit 2	−2.62114	0.01281
K09034	0047163	NDUFB10; NADH dehydrogenase (ubiquinone) 1 beta subcomplex subunit 10	−1.32268	4.45 × 10^−15^
K02132	0050561	ATPF1B, atpD; F-type H+-transporting ATPase subunit beta	−1.32455	4.6 × 10^−11^
**Fatty acid metabolism**				
K01962	0058323	accA; acetyl-CoA carboxylase carboxyl transferase subunit alpha	−1.15419	4.2 × 10^−8^
K02160	0053179	accB, bccP; acetyl-CoA carboxylase biotin carboxyl carrier protein	−1.16966	0.00244
K00645	0016765	fabD;	−9.43886	4.2 × 10^−45^
